# Prognostic Significance of Initial Serum Albumin and 24 Hour Daily Protein Excretion before Treatment in Multiple Myeloma

**DOI:** 10.1371/journal.pone.0128905

**Published:** 2015-06-08

**Authors:** Jia-Hong Chen, Shun-Neng Hsu, Tzu-Chuan Huang, Yi-Ying Wu, Chin Lin, Ping-Ying Chang, Yeu-Chin Chen, Ching-Liang Ho

**Affiliations:** 1 Division of Hematology-Oncology, Department of Medicine, Tri-Service General Hospital, National Defense Medical Center, Taipei, Taiwan; 2 Graduate Institute of Clinical Medicine, College of Medicine, Taipei Medical University, Taipei, Taiwan; 3 Division of Nephrology, Department of Medicine, Tri-Service General Hospital, National Defense Medical Center, Taipei, Taiwan; 4 Graduate Institute of Life Sciences, National Defense Medical Center, Taipei, Taiwan; 5 School of Public Health, National Defense Medical Center, Taipei, Taiwan

## Abstract

Renal failure is a common morbidity in multiple myeloma (MM). Although proteinuria has been increasingly reported in malignancies, it is not routinely used to refine risk estimates of survival outcomes in patients with MM. Here we aimed to investigate initial serum albumin and 24-hour daily protein excretion (24-h DPE) before treatment as prognostic factors in patients with MM. We conducted a retrospective analysis of 102 patients with myeloma who were ineligible for haematopoietic stem cell transplantation between October 2000 and December 2012. Initial proteinuria was assessed before treatment by quantitative analysis of 24-hour urine samples. The demographic and laboratory characteristics, survival outcome, and significance of pre-treatment 24-h DPE and albumin in the new staging system of MM were analyzed. Pre-treatment proteinuria (>300 mg/day) was present in 66 patients (64.7%). The optimal cut-off value of 24-h DPE before treatment was 500 mg/day. Analysis of the time-dependent area under the curve showed that the serum albumin and 24-h DPE before treatment were better than 24-h creatinine clearance rate and β2-microglobulin. A subgroup analysis showed that an initial excess proteinuria (24-h DPE ≥ 500 mg) was associated with poor survival status (17.51 vs. 34.24 months, p = 0.002). Furthermore, initial serum albumin was an independent risk factor on multivariate analysis (<2.8 vs. ≥2.8, hazard ratio = 0.486, p = 0.029). Using the A-DPE staging system, there was a significant survival difference among patients with stage I, II, and III MM (p < 0.001). Initial serum albumin and 24-h DPE before treatment showed significant prognostic factors in patients with MM, and the new A-DPE staging system may be utilized instead of the International Staging System. Its efficacy should be evaluated by further large prospective studies.

## Introduction

Multiple myeloma (MM) is a neoplastic disorder characterized by a single clone abundance of plasma cells occupying in the bone marrow and generating a monoclonal immunoglobulin, which sequentially results in end organ damage and related complications such as anemia, renal insufficiency, hypercalcaemia, skeletal events, and infection [1–4]. Although proteinuria is not uncommon in patients with malignancies, a high prevalence of proteinuria was reported in patients with MM [5–7]. Furthermore, the correlation between proteinuria and MM is well established, but its clinical impact on survival has not yet been elucidated owing to a lack of clinical studies.

Renal involvement in MM can manifest as subclinical proteinuria to overt proteinuria or even nephropathy. Proteinuria-induced renal failure remains a major cause of morbidity and mortality in patients with MM [8–10]. The earlier the magnitude of proteinuria is reduced, the lower the risks of renal disease progression and mortality become [11, 12]. Moreover, with the introduction of bortezomib-based treatment, survival has improved greatly in recent years when early improvement in renal function occurred [13–15]. The Durie Salmon (DS) staging system, which was primarily used 40 years previously for patients with MM, was designed with each stage divided into A and B subgroups according to renal function [16]. The International Staging System (ISS) published by the International Myeloma Working Group in 2005 introduced a new staging system using β2-microglobulin (β2M) and albumin levels as prognostic factors [17]. Whether the DS or ISS is used, renal function and albumin have been considered easy and good indicators of survival [18]. However, β2M is easily influenced by many factors including renal function, diverse autoimmune disease, and haematological malignancies [19–23]. Therefore, we try to introduce new parameters, including 24-h daily protein excretion (DPE) and albumin, to refine risk estimates of survival outcomes in patients with MM.

In this study, we investigated the initial serum albumin and 24-h DPE before treatment as prognostic factors in patients with MM who were ineligible to undergo haematopoietic stem cell transplantation (HSCT) and determined the significance of the new staging system of MM. We also evaluated the true incidence and clinical impact of proteinuria and other confounding factors on patients with MM.

## Patients and Methods

### Study population and diagnosis of MM

**Fig 1:**
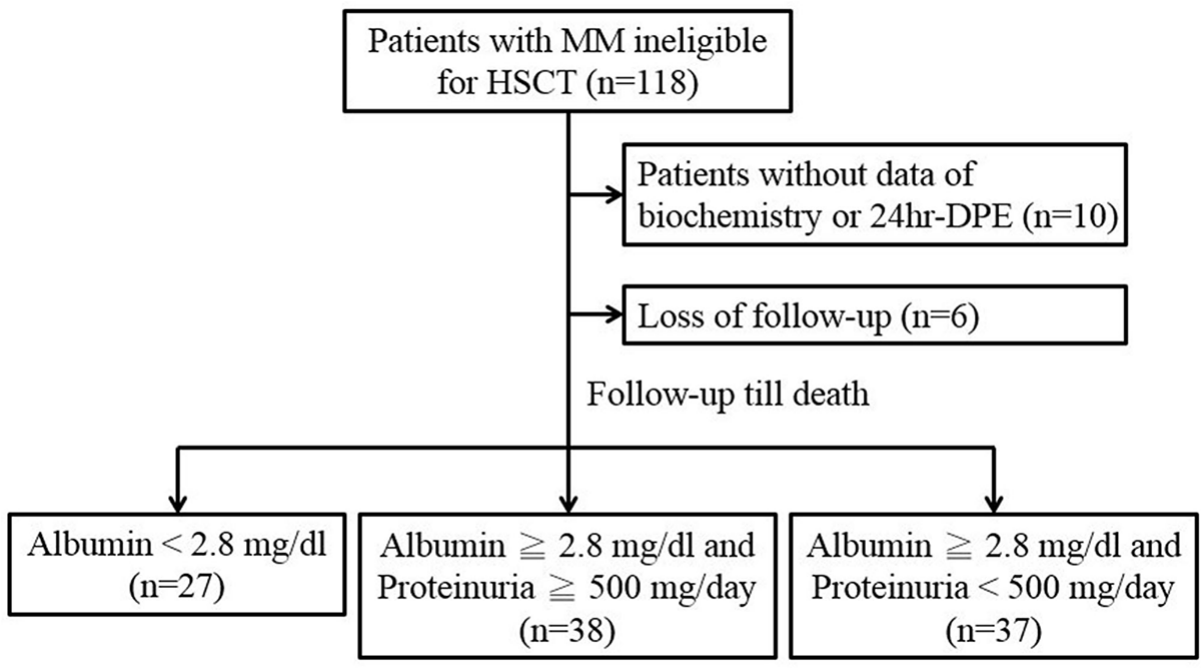
Patient classification.

This retrospective analysis enrolled a total of 102 patients with MM who were ineligible for HSCT in the Tri-Service General Hospital (TSGH) between October 2000 and December 2012 (Fig 1). Because the patient records/information were anonymized and de-identified prior to analysis in this study, informed consent was not required. The study was performed under the guidelines of the Helsinki Declaration and approved by the Human Subjects Protection Offices (institutional review board) of TSGH, National Defense Medical Center in Taiwan. All patients had symptomatic MM in accordance with the diagnostic criteria of the International Myeloma Working Group. All enrolled patients received treatment after the collection of 24-h urine samples for the measurement of creatinine clearance rate (Ccr) and DPE. The clinical information collected from the medical records included: age, sex, Eastern Cooperative Oncology Group performance status (ECOG PS) at diagnosis, disease severity, survival duration, and pre-treatment laboratory data, such as serum albumin, blood urea nitrogen (BUN), creatinine (Cr), total calcium, β2M, urinary 24-h Ccr, and 24-h DPE. The disease severity at diagnosis was defined by a different prognostic system [24, 25]. In addition, the impact of variable confounding factors on overall survival (OS) was analyzed in this study population.

### Assessment of initial albumin before treatment

Pre-treatment serum albumin was assessed by quantitative analysis of serum samples (g/dL). The demographic and laboratory characteristics, prevalence, survival outcome, and significance of pre-treatment serum albumin in the MM staging system were analyzed.

### Assessment of initial proteinuria before treatment

Proteinuria was defined as excreting > 300 mg of total protein per 24 hours (24-h DPE > 300 mg) according to the Kidney Disease Outcomes Quality Initiative guidelines [26]. Quantitative analysis data of the 24-h urine samples before treatment were analyzed to ascertain the DPE and Ccr values. Urinary Cr and protein concentrations were determined using Jaffe and colorimetric methods, respectively. The total urinary protein (mg/day) was determined by multiplying the total urine volume (dL) by the protein concentration in the test sample (mg/dL).

### MM treatment protocol

Patients with MM who were ineligible for the HSCT received various treatments; they could be divided into three groups: those who received conservative treatment (no treatment or dexamethasone 20 mg/m^2^ orally only), those who received conventional chemotherapy and thalidomide (e.g., MP, VAD, TD, and TMD), and those who received novel agents with bortezomib-based treatment (BTD).

The MP regimen consisted of melphalan (8 mg/m^2^) and prednisolone (60 mg/m^2^) for 4 days; the VAD regimen consisted of vincristine (0.4 mg) and doxorubicin (9 mg/m^2^) for 4 days and dexamethasone (40 mg) for 12 days; and the TD regimen consisted of 200 mg thalidomide orally per day plus 5–10 mg dexamethasone intravenously 4 times per day. The BTD arm was identical to the TD arm plus bortezomib 1.3 mg/m^2^ on days 1, 4, 8, and 11 of each cycle.

### Statistical analysis

Data were analyzed using SPSS. In addition to descriptive and frequency statistics, univariate and multivariate analyses were used to evaluate the significance of all confounding factors in the patients with MM. To further evaluate the predictive ability of initial serum albumin, β2M, 24-h Ccr, and 24-h DPE, we conducted a time-dependent receiver operating characteristic curve (ROC) analysis for censored records. This analysis uses sensitivity and specificity, both of which are time-dependent, to measure the prognostic capacity of a survival model as measured by the area under the curve (AUC) [27]. Compared with survival status, the Kaplan-Meier curve was used to present their differences and the log-rank test was used to analyze the data. We considered p-values < 0.05 as significant for all analyses.

## Results

### Patient characteristics

Since October 2000, a total of 102 patients (median age, 71.0 years; median OS, 18.0 months) with MM were recruited in this study. The median survival time of stage I, II, III by ISS staging system were 67, 25 and 15 months (P = 0.049). On average, myeloma affects male patients more often than female patients at a ratio of 1.61:1. Table 1 summarizes the patients’ baseline characteristics, myeloma types, and survival duration. A total of 56 patients (54.9%) were diagnosed with the immunoglobulin G (IgG) type myeloma, followed by the light chain type (19.6%) and immunoglobulin A (IgA) type (18.6%). Only 7 patients (6.9%) had the non-secretory type of myeloma. However, 27 patients (26.5%) had renal function impairment (serum Cr ≥ 2.0 mg/dL), among which 12 (44%) were associated with light chain type myeloma, 8 (30%) with IgG type, 6 (22%) with IgA type, and 1 (4%) with non-secretory type. Sixty-six of the 102 patients (64.7%) had proteinuria prior to treatment. A total of 40 patients (39.2%) received BTD in our study population.

**Table 1:** Baseline patient characteristics, myeloma types, and survival.

Characteristic	Patients, N = 102
Median age, years	71 (40–92)
Current status (n, %)	
Alive	27 (26.5%)
Sex (n, %)	
Male	63 (61.8%)
Female	39 (38.2%)
ECOG PS	
1–2	67 (65.7%)
3–4	35 (34.3%)
Myeloma type (n, %)	
IgG	56 (54.9%)
IgA	19 (18.6%)
Light chain	20 (19.6%)
Non-secretory	7 (6.9%)
Disease stage at diagnosis (ISS; n, %)	
Stage I	18 (17.6%)
Stage II	34 (33.3%)
Stage III	50 (49.0%)
Renal insufficiency at diagnosis (n, %)^a^	27 (26.5%)
Proteinuria^b^	66 (64.7%)
Hypocalcaemia at diagnosis^c^	29 (28.4%)
Bortezomib therapy	40 (39.2%)
Mean overall survival (months)	25.2
Median overall survival (months)	18.0

ECOG PS, Eastern Cooperative Oncology Group performance status; IgG, immunoglobulin G; IgA, immunoglobulin A; ISS, International Staging System

^a^Serum creatinine ≥ 2 mg/dL

^b^24-h daily protein excretion > 300 (mg/day)

^c^Total calcium > 10 mg/dL

### Time-dependent ROC analysis and the impact of proteinuria

**Fig 2:**
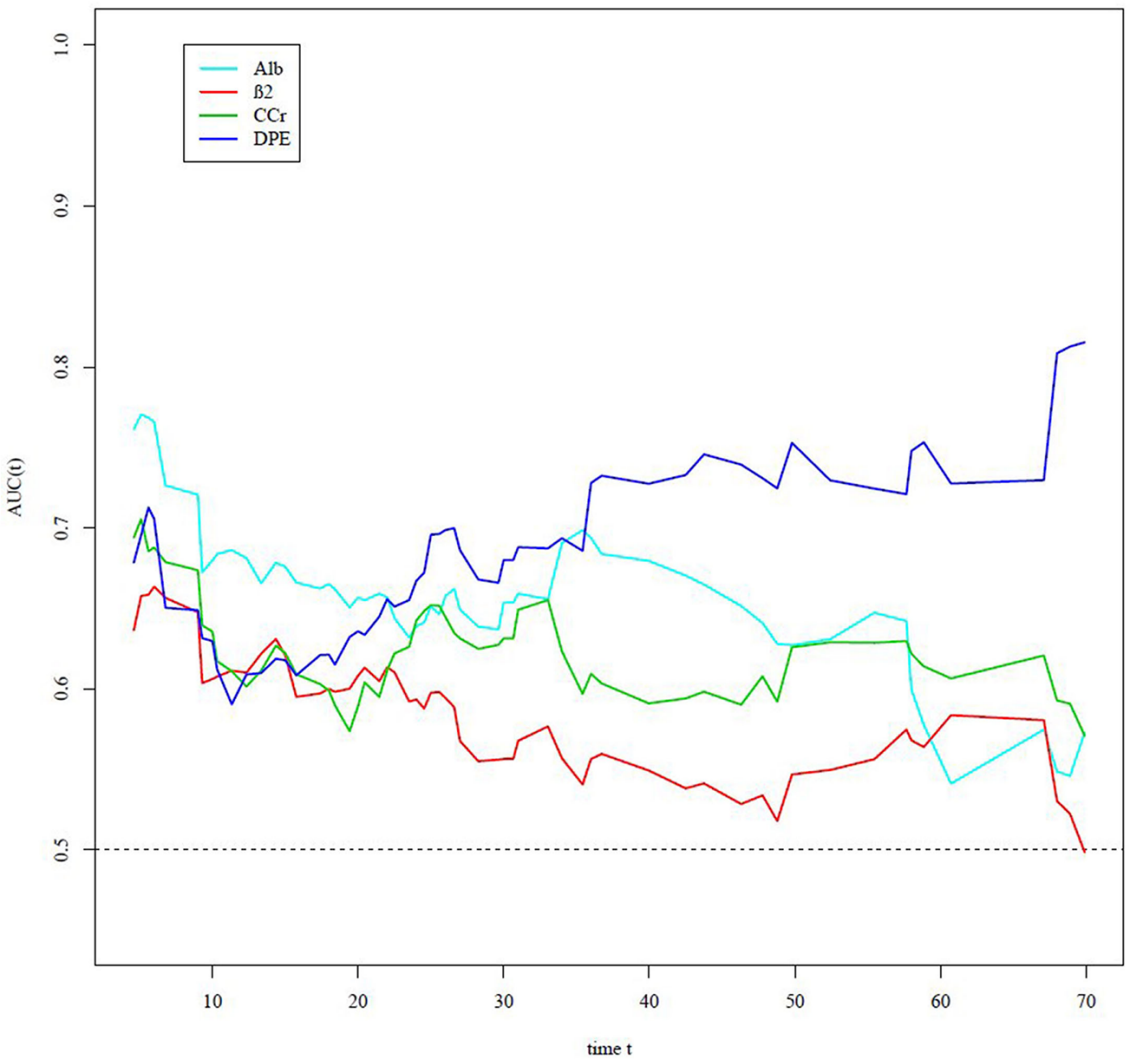
Time-dependent areas under the curve.

In our study, the parameters associated with renal function include serum β2M, albumin, 24-h Ccr, and 24-h DPE. The optimal cut-off value of the initial 24-h DPE was 500 mg/day on ROC analysis. According to time-dependent AUC analysis (Fig 2), the prognostic capacity of initial serum albumin and 24-h DPE before treatment was better than those of 24-h Ccr and β2M. In addition, the pre-treatment albumin level played the most important role in survival capacity, especially within 22 months after anti-myeloma treatment.

On subgroup univariate analysis using the optimal cut-off value of proteinuria (500 mg/day), patients with initial excess proteinuria (≥500 mg/day) had a significantly poorer ECOG PS (p = 0.018), higher serum BUN (p = 0.004), higher serum Cr (p = 0.020), lower serum albumin levels (p = 0.009), and shorter survival duration (17.51 vs. 34.24 months, p = 0.002) (Table 2).

**Table 2:** Univariate analysis of factors associated with proteinuria in multiple myeloma.

Patient characteristics	24-h DPE	24-h DPE	P-value
	>500 mg/day	<500 mg/day	
	(n = 55)	(n = 47)	
Sex (M:F ratio)	1:0.62	1:0.62	NS
Age, years	70.29±10.85	68.64±11.10	NS
ECOG PS	2.33±0.98	1.85±1.01	0.018
ISS stage	2.42±0.79	2.20±0.72	NS
24-h Ccr (mL/min)	53.70±47.18	63.69±37.71	NS
BUN (mg/dL)	41.07±35.46	25.43±15.28	0.004
Cr (mg/dL)	2.56±2.85	1.47±1.68	0.020
T Ca (mg/dL)	9.71±1.89	9.82±1.39	NS
F Ca (mg/dL)	4.95±1.11	5.17±0.89	NS
Albumin (g/dL)	3.15±0.75	3.29±0.64	0.009
β2M	11.25±14.89	8.24±11.23	NS
Effect of bortezomib	19 (34.55%)	21 (44.68%)	NS
Survival time	17.51±17.05	34.24±32.16	0.002

24-h DPE, 24-hour daily protein excretion; NS, not significant; ECOG PS, Eastern Cooperative Oncology Group performance status; ISS, International Staging System; Ccr, creatinine clearance rate; BUN, blood urea nitrogen; T Ca, serum total calcium; F Ca, serum free calcium; β2M, β2-microglobulin

Data are means ± SD or percentages.

### Analysis of all confounding factors on OS

Subsequent analysis of all variables associated with all-cause mortality was performed by univariate and multivariate analyses. The univariate analysis of the continuous method showed that age, ECOG PS, ISS stage, proteinuria, 24-h Ccr, serum BUN, total calcium, and albumin were significant prognostic factors (Table 3). Using the dichotomous method, the results revealed that ECOG PS, stage ISS, proteinuria, 24-h Ccr, serum BUN, Cr, total calcium, albumin, and the effect of bortezomib were significant. However, serum β2M was not significant on univariate analysis using both the continuous and dichotomous methods.

**Table 3:** Hazard ratio on univariate analysis.

	Continuous			Dichotomous			
	HR	95% CI	P value	Cut-off value	HR	95% CI	P value
**Patient characteristic**							
Sex				Female	1.000		
				Male	0.983	0.657–1.471	NS
Age, years	1.019	1.001–1.038	0.036	<60	1.000		
				≥60	1.384	0.825–2.320	NS
ECOG PS	1.419	1.214–1.829	<0.001	0–2	1.000		
				3–4	1.691	1.113–2.570	0.014
ISS stage	1.358	1.039–1.774	0.025	1	1.000		
				2	1.196	0.667–2.146	NS
				3	1.774	1.027–3.066	0.040
**Laboratory data**							
24-h DPE	1.000	1.000–1.000	0.009	<500	1.000		
				≥500	2.021	1.323–3.087	0.001
24-h Ccr (mL/min)	0.995	0.990–1.000	0.048	< 45	1.000		
				≥45	0.590	0.393–0.885	0.011
BUN (mg/dL)	1.012	1.005–1.019	0.001	< 20	1.000		
				≥20	1.808	1.183–2.763	0.006
Cr (mg/dL)	1.041	0.971–1.116	NS	<2	1.000		
				≥2	1.919	1.217–3.027	0.005
T Ca (mg/dL)	1.142	1.002–1.302	0.047	<10	1.000		
				≥10	1.637	1.055–2.539	0.028
F Ca (mg/dL)	0.937	0.740–1.185	NS	<4.5	1.000		
				≥4.5	1.213	0.682–2.159	NS
Albumin (g/dL)	0.627	0.466–0.844	0.002	<2.8	1.000		
				≥2.8	0.376	0.235–0.603	<0.001
β2M	1.004	0.991–1.018	NS	<5.5	1.000		
				≥5.5	1.045	0.705–1.549	NS
Effect of bortezomib				Without	1.000		
				With	0.451	0.298–0.682	<0.001

HR, hazard risk of death; CI, confidence interval; NS, not significant; ECOG PS, Eastern Cooperative Oncology Group performance status; ISS, International Staging System;

24-h DPE, 24-hour daily protein excretion; 24-h Ccr, 24-hour creatinine clearance rate; BUN, blood urea nitrogen; Cr, creatinine; T Ca, serum total calcium; F Ca, serum free calcium; β2M, β2-microglobulin

Further multivariate analysis of the prognostic factors using the Cox proportional hazard model showed that only serum BUN and Cr were significant prognostic factors on the continuous method. Using the dichotomous method, however, the result revealed that pre-treatment serum albumin (<2.8 mg/dL) was an independent prognostic factor (<2.8 vs. ≥2.8; hazard ratio, 0.486; 95% confidence interval, 0.254–0.930; p = 0.029). Similarly, serum β2M was not significant in multivariate analysis by using both the continuous and dichotomous methods (Table 4).

**Table 4:** Multivariate analysis on a Cox proportional hazard model.

	Continuous			Dichotomous			
	HR	95% CI	P value	Cut-off value	HR	95% CI	P value
**Patient characteristic**							
Sex	1.451	0.797–2.640	NS	Female	1.000		
				Male	0.940	0.505–1.750	NS
Age, years	0.996	0.966–1.027	NS	<60	1.000		
				≥60	1.015	0.385–2.676	NS
ECOG PS	1.203	0.803–1.801	NS	0–2	1.000		
				3–4	1.697	0.806–3.577	NS
Stage ISS	1.060	0.637–1.761	NS	1	1.000		
				2	1.153	0.323–4.115	NS
				3	2.535	0.513–12.514	NS
**Laboratory data**							
24-h DPE	1.000	1.000–1.000	NS	<500	1.000		
				≥500	1.442	0.681–3.054	NS
24-h Ccr (mL/min)	0.995	0.985–1.006	NS	<45	1.000		
				≥45	1.710	0.676–4.329	NS
BUN (mg/dL)	1.026	1.005–1.047	0.014	<20	1.000		
				≥20	1.070	0.476–2.407	NS
Cr (mg/dL)	0.712	0.537–0.942	0.018	<2	1.000		
				≥2	2.550	0.949–6.849	NS
T Ca (mg/dL)	1.039	0.842–1.282	NS	<10	1.000		
				≥10	0.989	0.477–2.050	NS
F Ca (mg/dL)	0.753	0.521–1.088	NS	<4.5	1.000		
				≥4.5	1.116	0.507–2.459	NS
Albumin (g/dL)	0.688	0.442–1.073	NS	<2.8	1.000		
				≥2.8	0.486	0.254–0.930	0.029
β2M	1.035	0.979–1.094	NS	<5.5	1.000		
				≥5.5	0.433	0.121–1.549	NS
Effect of bortezomib	0.467	0.216–1.009	NS	Without	1.000		
				With	0.581	0.285–1.186	NS

HR, hazard risk of death; CI, confidence interval; NS, not significant; ECOG PS, Eastern Cooperative Oncology Group performance status; ISS, International Staging System;

24-h DPE, 24-hour daily protein excretion; 24-h Ccr, 24-hour creatinine clearance rate; BUN, blood urea nitrogen; Cr, creatinine; T Ca, serum total calcium; F Ca, serum free calcium; β2M, β2-microglobulin

### A-DPE staging system

**Fig 3:**
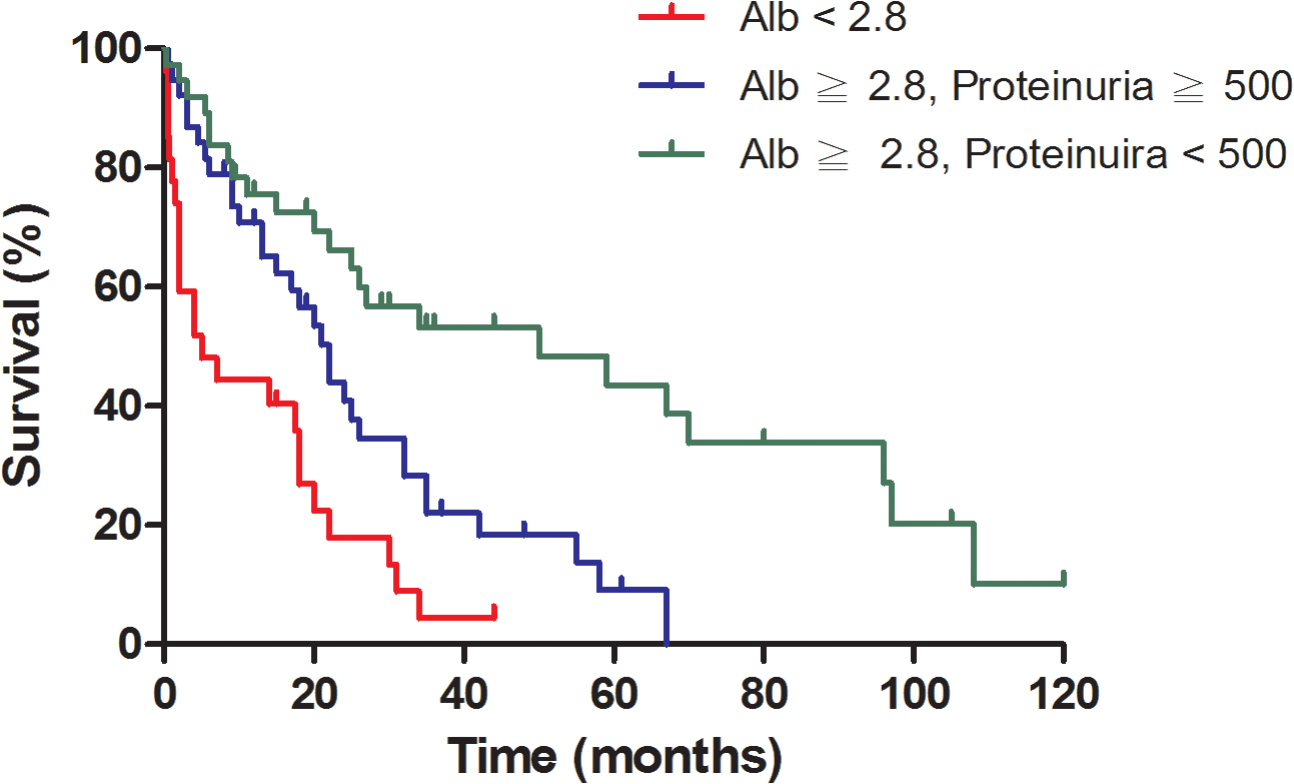
Overall survival of multiple myeloma based on clinical stage at diagnosis by A-DPE staging.

According to the results of time-dependent AUC, multivariate, and univariate analyses, we formulated a new experimental staging system, the A-DPE staging system, based on initial serum albumin and 24-h DPE prior to treatment. In this system, stage I defined as albumin ≥ 2.8 g/dL and 24-h DPE < 500 mg/dL; stage II as albumin ≥ 2.8 g/dL and 24-h DPE ≥ 500 mg/dL; and stage III as albumin < 2.8 g/dL. Applying this system, we found that 37 patients had stage I, 38 had stage II, and 27 had stage III. According to this staging system, there was a significant survival difference among myeloma patients with stage I, II, and III disease (p < 0.001, Fig 3).

## Discussion

In patients with MM, the significance of initial serum albumin and 24-h DPE before treatment led to the introduction of the new A-DPE staging system, and we found a significant survival difference among the different stages (p < 0.001). In addition, our results indicate that 64.7% (66/102) of the patients had proteinuria, which was not uncommon as previous studies revealed. It also demonstrated that patients with excess proteinuria (24-h DPE ≥ 500 mg/day) tended to have poor survival status (17.51 vs. 34.24 months, p = 0.002) and that those with lower albumin levels (<2.8 g/dL, A-DPE stage III) had the worst survival rate (p < 0.001). To date, our study was the first to investigate the impact and prevalence of proteinuria and significance of 24-h DPE in patients with MM. These findings are important because the current clinical practice for predicting outcomes in patients with MM are solely based on serum albumin and β2M without explicit consideration of the severity of concomitant proteinuria.

Proteinuria, a marker of kidney disease, is strongly associated with a risk of adverse outcomes [28–30]. Many studies have demonstrated that the prevalence of proteinuria increased with poor control of blood pressure, a high glycated haemoglobin level, and accompanying renal or malignant disease [6, 29, 31]. In addition, patients with malignancies often present with proteinuria but without significant symptoms to emphasize its clinical importance. Thus, we use quantitative analysis of 24-h urine to ascertain DPE and renal function. Despite its evolving role as a major risk factor of all-cause mortality, little is known about the clinical impact of proteinuria in patients with MM. In an attempt to choose a more significant prognostic factor and determine the correlation between 24-h DPE and kidney function, we were unable to find any study that included 24-h DPE as a prognostic factor. Thus, to our knowledge, this study is the first to investigate serum albumin and 24-h DPE as prognostic factors in patients with MM. Furthermore, the parameters associated with renal function include not only albumin and 24-h DPE but also serum β2M, and 24-h Ccr. Instead of β2M, our new staging system used 24-h DPE owing to β2M not being significant if kidney function was relatively normal. Of note, our study proved that serum β2M was not significant on univariate or multivariate analysis using both the continuous and dichotomous methods. Thus, the effect of β2M on survival outcomes might be mainly owing to declining kidney function.

On time-dependent AUC analysis, the results indicate that 24-h DPE is a new potential prognostic factor in patients with MM, especially for long-term survival outcomes. Moreover, it demonstrates that pre-treatment serum albumin has the better predictive value in the early course of the disease, whereas pre-treatment 24-h DPE has better predictive value in the late course of the disease. In addition, multivariate analysis of the prognostic factors revealed that pre-treatment serum albumin was the most important independent risk factor of all-cause mortality. Thus, we incorporate pre-treatment serum albumin and 24-h DPE into a discrete new staging system for predicting outcomes in patients with MM. Survival curves of different stages were discrete; they were able to show that 24-h DPE was a more significant and powerful prognostic factor than β2M.

Recent studies showed that bortezomib appears to be most valuable in this setting of treatment with MM and renal impairment and that, when combined with other drugs, increases the probability of rapid remission and related improvements in renal function [32]. Substantial evidence indicates that the administration of bortezomib and high-dose dexamethasone with or without a third drug (e.g., cyclophosphamide, thalidomide, or doxorubicin) is an appropriate option for patients with any degree of renal impairment [15, 33]. In the future, large, prospective cohort studies may be needed to confirm whether the bortezomib-based regimen could overcome poor prognosis in the presence of any degree of renal impairment or proteinuria.

However, our study had some inherent limitations. First, because of its retrospective design and small sample size, patient heterogeneity, and different treatment protocols, the prognostic impact of proteinuria on patients with MM needs to be confirmed by large, prospective cohort studies. Second, the prevalence of proteinuria may be underestimated because we only enrolled patients for whom a 24-h DPE and chart were available may create some selection bias. Third, the majority of enrolled patients did not undergo renal biopsy to clarify whether the proteinuria was caused solely by paraneoplastic syndrome or other systemic disorders that involve the kidneys. Fourth, the information of genetic abnormalities was lacking because of the lack of a routine to perform cytogenetic studies and equipment to perform fluorescent in situ hybridisation in our institute. Therefore, it was difficult to identify who had high-risk genetic factors, which may affect the choice of frontline treatment and impair survival outcomes. Fifth, few patients received newer therapeutic strategies such as frontline bortezomib–containing regimens. Only 3 patients received bortezomib as the first line treatment since June 2012. Finally, the follow-up duration was too short to evaluate the survival benefit of treatment.

In summary, here we demonstrated that the prevalence of proteinuria is not uncommon in patients with MM. In addition, we found that the initial 24-h DPE combined with serum albumin before treatment possess powerful and significant prognostic value for predicting survival outcomes in patients with MM. Routine screening of pre-treatment proteinuria in patients with MM may help clinicians provide better patient care. Whether reducing proteinuria improves survival requires further investigations.

## Supporting Information

S1 Fig According to the calculation of AUC by time, and added the calculated figures. (PDF)

S2 Fig According to the calculation of AUC by time, and added the calculated figures. (PDF)

S3 Fig Overall survival of multiple myeloma based on clinical stage at diagnosis by ISS staging. (TIF)

S4 Fig Certificate of English editing. (PDF)

S5 Fig Institutional review board. (PDF)
