# Supplementary figures and images for: Prognostic Significance of Initial Serum Albumin and 24 Hour Daily Protein Excretion before Treatment in Multiple Myeloma
Source: PLoS One. 2015 Jun 8;10(6):e0128905. doi: 10.1371/journal.pone.0128905 (PMC4459796; doi:10.1371/journal.pone.0128905)

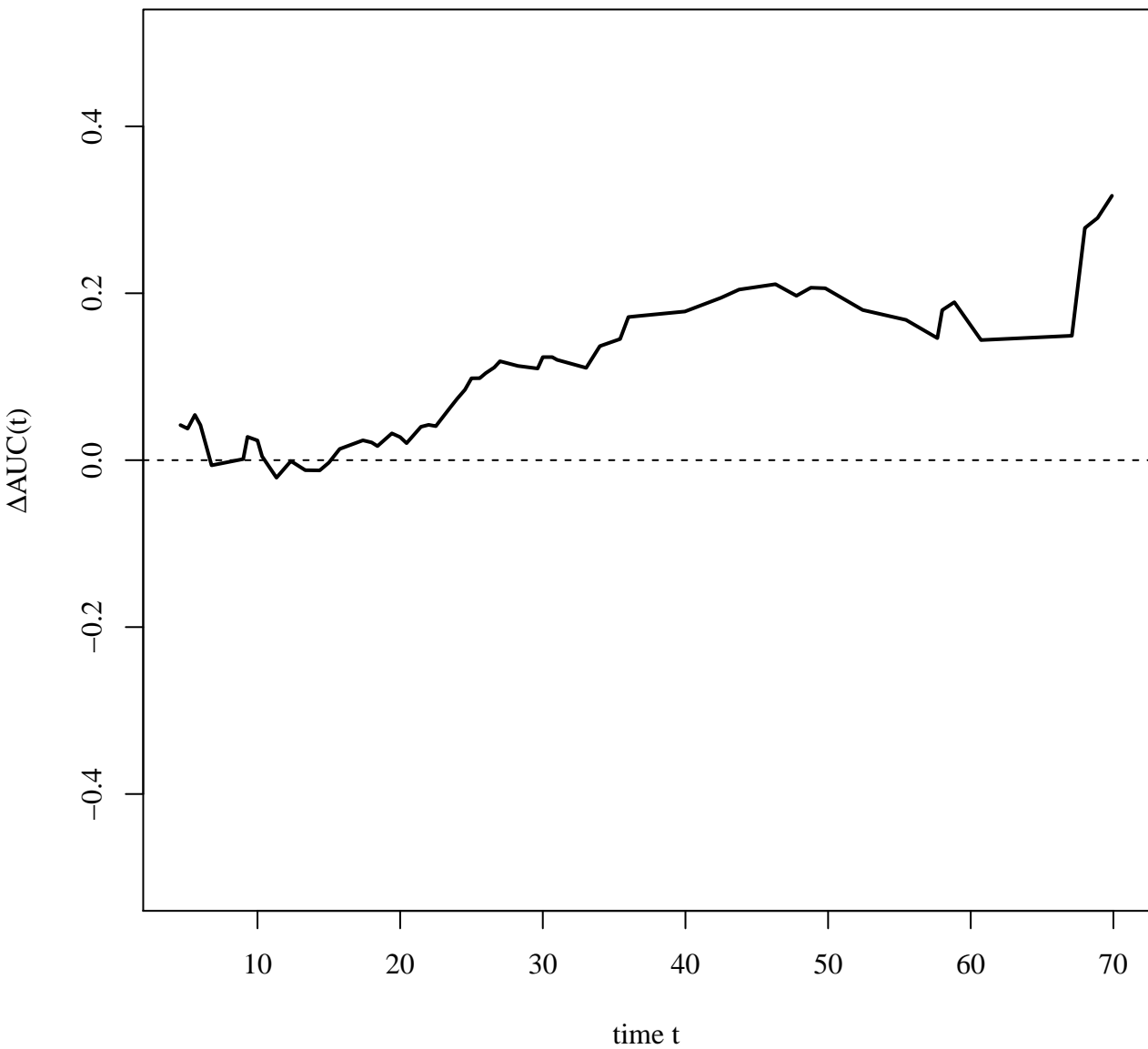

Supplement: S1 Fig — (PDF) [file pone.0128905.s001.pdf]

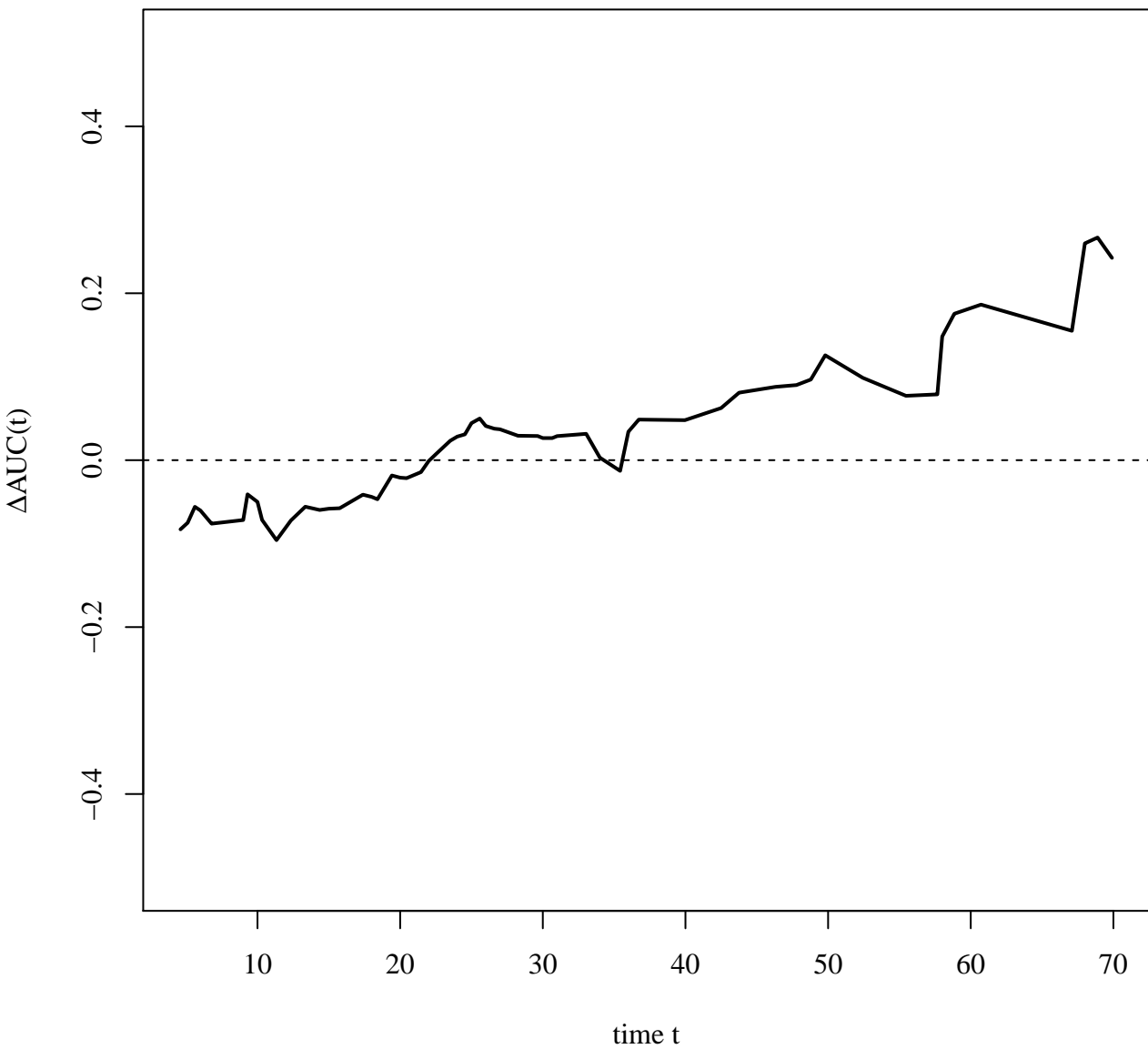

Supplement: S2 Fig — (PDF) [file pone.0128905.s002.pdf]

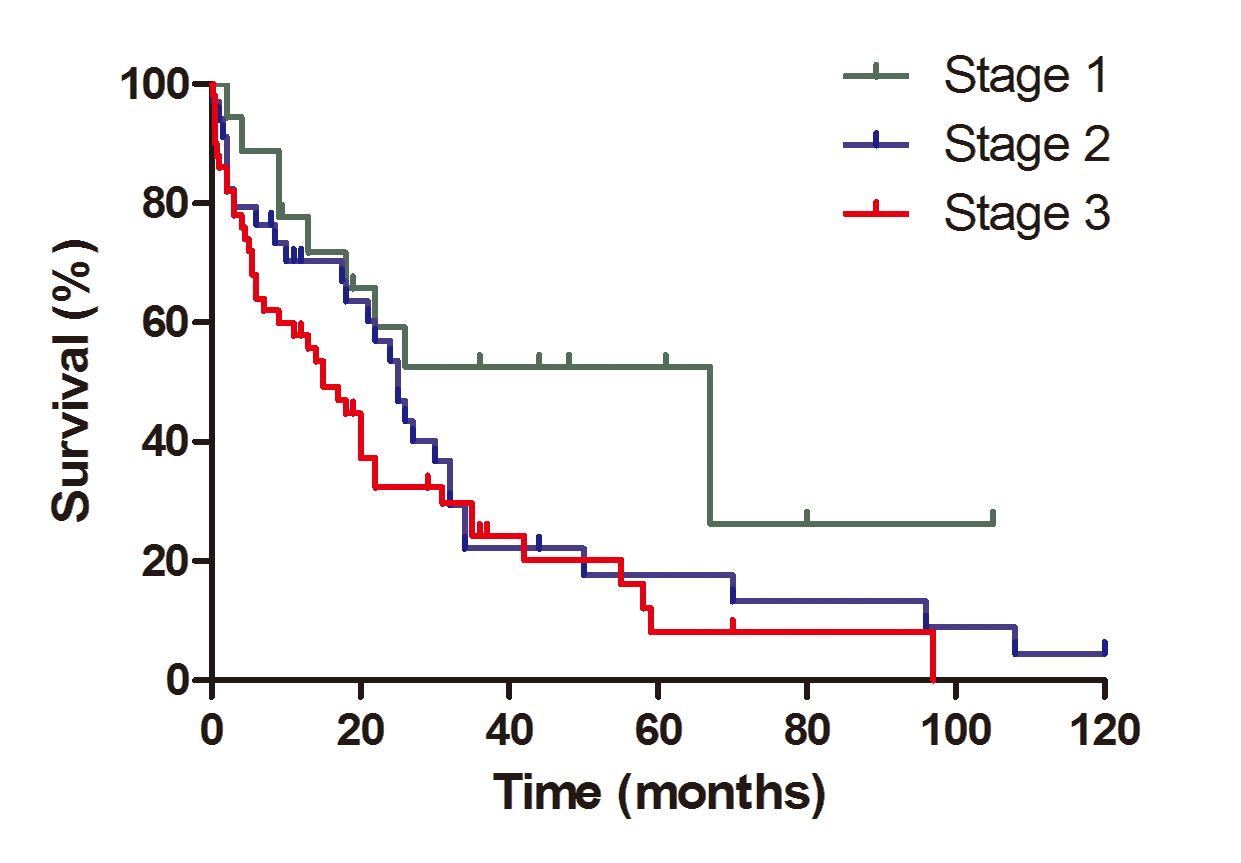

Supplement: S3 Fig — (TIF) [file pone.0128905.s003.tif]
