# Supplementary material for: Prognostic Significance of Initial Serum Albumin and 24 Hour Daily Protein Excretion before Treatment in Multiple Myeloma
Source: PLoS One. 2015 Jun 8;10(6):e0128905. doi: 10.1371/journal.pone.0128905 (PMC4459796; doi:10.1371/journal.pone.0128905)
Supplement: S4 Fig — (PDF) [file pone.0128905.s004.pdf]

CERTIFICATE OF ENGLISH EDITING

This document certifies that the paper listed below has been edited to ensure that the language is clear and free of errors. The edit was performed by professional editors at Editage, a division of Cactus Communications.The intent of the author's message was not altered in any way during the editing process. The quality of the edit has been guaranteed, with the assumption that our suggested changes have been accepted and have not been further altered without the knowledge of our editors.

Title Of The Paper

Prognostic Significance of Initial Serum Albumin and 24 Hour Daily Protein Excretion Before Treatment in Multiple Myeloma

Authors

Jia-Hong Chen, Shun-Neng Hsu, Tzu-Chuan Huang, Yi-Ying Wu, Chin Lin, Ping-Ying Chang, Yeu-Chin Chen, Ching-Liang Ho

Job Code

YDFSF_2

Signature

Nikesh Gosalia,
Vice President, Author Services, Editage

Date of Issue
April 23, 2015

Editage, a brand of Cactus Communications, offers professional English language editing and publication support services to authors engaged in over 500 areas of research. Through its community of experienced editors, which includes doctors, engineers, published scientists, and researchers with peer review experience, Editage has successfully helped authors get published in internationally reputed journals. Authors who work with Editage are guaranteed excellent language quality and timely delivery.

Contact Editage

Worldwide	Japan	Korea	China	Brazil	Taiwan
request@editage.com	submissions@editage.com	submit-korea@editage.com	fabiao@editage.cn	inquiry.brazil@editage.com	submitjobs@editage.com
+1 877-334-8243	+81 03-6868-3348	1544-9241	400-005-6055	0800-892-20-97	02 2657 0306
www.editage.com	www.editage.jp	www.editage.co.kr	www.editage.cn	www.editage.com.br	www.editage.com.tw
