# Supplementary material for: Prognostic Significance of Initial Serum Albumin and 24 Hour Daily Protein Excretion before Treatment in Multiple Myeloma
Source: PLoS One. 2015 Jun 8;10(6):e0128905. doi: 10.1371/journal.pone.0128905 (PMC4459796; doi:10.1371/journal.pone.0128905)
Supplement: S5 Fig — (PDF) [file pone.0128905.s005.pdf]

Tel：886-2-87923311 ext 10552
Fax：886-2-2793-6049
E-mail：tsghirb@ndmctsgh.edu.tw

國防醫學院三軍總醫院
人體試驗審議會

台北市 11490 內湖區成功路二段
325 號醫療大樓五樓 5113 室
No.325,Sec.2, Cheng-Kung Rd.
Neihu 11490, Taipei, Taiwan, R.O.C

人體試驗計畫同意函

查血液腫瘤科陳佳宏醫師主持之「研究骨橋蛋白(osteopontin)和 nodal 表現在多發性骨髓瘤的角色」（TSGHIRB 核准編號：2-102-05-107）研究計畫案，業經本院 2013 年 11 月 27 日人體試驗審議會第二審議會第 170 次會議審查通過但須修正，並於 2013 年 12 月 25 日經執行秘書審核通過同意執行。該計畫案經評估屬低度風險，**（持續審查頻率為每年一次）**，有效期限至 2014 年 12 月 24 日，特此證明。

計畫主持人應於同意函有效期屆滿前一個月提出展延申請，本案須經本院人體試驗審議會通過後，方可繼續執行。

人體試驗審議會召集人
余慕賢

Letter of Approval
Tri-Service General Hospital National Defense Medical Center

Date of approval: 12/25/2013

TSGHIRB No.：2-102-05-107
Protocol Title：Study the role of osteopontin and nodal expression in multiple myeloma
Principal Investigator：Dr. Jia-Hong Chen
Protocol No.：2-102-05-107
Protocol Version：v3.0_2013/12/19
Informed Consent Form：V4.0_2013/12/19
Other documents：Chinese Abstract：V3.0_2013/11/21

On 12/25/2013, the Institutional Review Board Ⅱ of the Tri-Service General Hospital, National Defense Medical Center decided to approve the above-named application. According to the written operating procedures, GCP, and the applicable regulatory requirements, this application is approved by the Institutional Review Board of TSGHIRB. The board is organized under, and operates per International Conference on Harmonization (ICH) / WHO Good Clinical Practice (GCP) and the applicable laws and regulations.
This approval is valid for 1 year till 12/24/2014 The principal investigator is required to submit the application for extension 1 month before the expiration date.
Sincerely,
Mu-Hsien Yu,M.D., Ph.D.
Yu Mu Hsien

Chairman
Institutional Review Board

三軍總醫院 TSGHIRB 人體試驗審議會
